# The Goldilocks zone in neural circuits

**DOI:** 10.7554/eLife.22735

**Published:** 2016-12-02

**Authors:** Mark D Humphries

**Affiliations:** Faculty of Biology, Medicine and Health, University of Manchester, Manchester, United Kingdommark.humphries@manchester.ac.uk

**Keywords:** motor networks, lognormal, spinal cord, motor control, neuronal ensemble, CPG, Other

## Abstract

How do networks of neurons remain both stable and sensitive to new inputs?

**Related research article** Petersen, PC, Berg RW. 2016. Lognormal firing rate distribution reveals prominent fluctuation-driven regime in spinal motor networks. *eLife* **5**:e18805. doi: 10.7554/eLife.18805

Networks of neurons are tough beasts to control. If too many of the connections between the neurons are excitatory, the network becomes hyperactive, driven by feedback from neuron to neuron. But if too many of the connections are inhibitory, the network goes silent, save for a few blips of activity, mercilessly crushed by inhibition. So how do real neuronal networks stay in the Goldilocks zone between too quiet and too loud? How do they maintain stable activity yet remain sensitive to new inputs?

One clue comes from the long-tailed distributions of spiking rates that are seen throughout the cortex: a small number of neurons respond strongly to a specific input, but most spike only weakly, and thus remain ready to spike again in response to a new input (Wohrer et al., 2013; Buzsáki and Mizuseki, 2014). Another clue comes from the balanced network model: according to this model the excitatory and inhibitory inputs to cortical neurons cancel on average, so the neurons can maintain stable, irregular activity (van Vreeswijk and Sompolinsky, 1996; Renart et al., 2010; see Figure 1A,B). However, we don't know how these two clues fit together to explain how neuronal networks reach the Goldilocks zone: in particular, can balanced networks produce long-tailed distributions of spiking rates?

**Figure 1.**
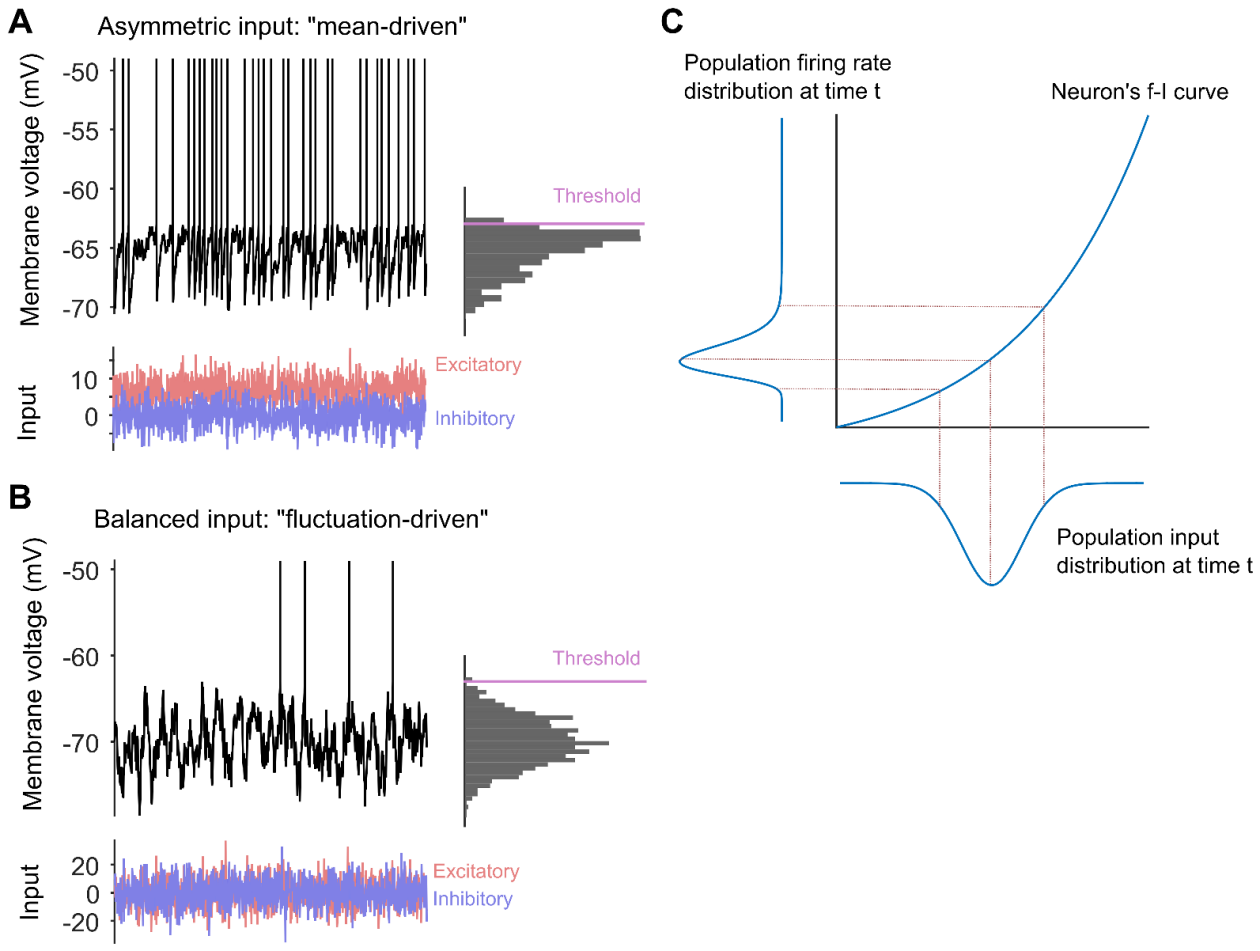
Long-tailed distributions and balanced networks. (**A**) When a single neuron receives more excitatory input (red) than inhibitory input (blue), its membrane voltage (black line) spikes regularly. In this "mean-driven" model the membrane voltage spends much of its time at or near the spiking threshold (see histogram on right), and spiking is driven by the difference between the means of the excitatory and inhibitory inputs. However, most neurons do not spike as rapidly or regularly as predicted by the "mean-driven" model. (**B**) The theory of balanced networks proposes that the inhibitory and excitatory inputs to a neuron have approximately the same mean and the same variance. Consequently, spiking is driven by random fluctuations in the inputs, when the excitatory input briefly exceeds the inhibitory input. A signature of this “fluctuation-driven” regime is that the distribution of the membrane voltage is approximately Gaussian (histogram). Fluctuation-driven neurons spike slowly and irregularly, similar to the majority of the neurons in the cortex. (**C**) The relationship between the input to a neuron (blue curve below the x-axis) and its spiking or firing rate (blue curve to the left of the y-axis) is described by a firing rate versus input (*f–i*) curve. In 2011 Roxin et al. predicted that an expansive *f–i* curve (see main text) would convert a Gaussian input into a long-tailed distribution of firing rates in the fluctuation-driven regime. Moreover, according to this theory, if each neuron has a Gaussian input, then the overall population of neurons will also have a Gaussian input, and if each neuron has an expansive *f–i* curve in the fluctuation-driven regime, then the output of the population will be long-tailed.

Now, in eLife, Peter Petersen and Rune Berg of the University of Copenhagen report compelling experimental evidence that they can (Petersen and Berg, 2016; Figure 1C). They did this by testing a theoretical model that was published in 2011 (Roxin et al., 2011). The experiments were performed on the spinal network in turtles and combined intracellular and large-scale extracellular recordings of neural activity. The recordings were made during periods of evoked motor behaviour (that is, when the turtle was scratching itself).

Petersen and Berg first showed that the spinal network was balanced. They identified individual neurons in the spinal cord that existed in a regime called the "fluctuation-driven" regime that is characteristic of balanced networks. The membrane voltages of these neurons fluctuated widely between spikes, as expected from neurons receiving the same inhibitory and excitatory input on average (Figure 1B). They also identified neurons that existed in the "mean-driven" regime (Figure 1A): in these neurons the membrane voltages moved from low values to high values rapidly and directly following each spike. Petersen and Berg then showed that it was possible to switch between the fluctuation-driven and mean-driven regimes by changing the balance between the excitatory and inhibitory inputs to the neurons.

Next they tested a slightly off-the-wall prediction made by Alex Roxin and co-workers for neurons in the fluctuation-driven regime (Roxin et al., 2011). This work predicted that if these neurons have an expansive output curve – that is, if the output increases faster than linear as the input increases – then their output spike-rate will have a long-tailed distribution (Figure 1C). Unexpectedly, Petersen and Berg showed that their fluctuation-driven neurons all had such an expansive output curve.

Petersen and Berg then used large-scale population recordings to address the key question: do these expansive output curves give rise to the predicted long-tailed distribution of firing rates across a network? The answer was a resounding yes. The populations they recorded had lognormal distributions of firing rates, and the neurons within each population sat on a continuum between the fluctuation- and mean-driven regimes. Intriguingly, their data suggest that these regimes bore no relation to whether the neurons were inter- or motor-neurons.

Petersen and Berg's work is a rarity in systems neuroscience, an experimental study that tests a computational theory directly, and exhaustively. They have provided compelling evidence that a combination of balanced input and expansive output can hold a network in the Goldilocks zones (that is, keep it both stable and responsive). And by working in the spinal cord networks of the turtle, they were able to show that all these properties exist during ongoing behaviour, and not just during spontaneous neuronal activity. Moreover, they remind us there is nothing privileged about the dynamics of cortical circuits, or the dynamics of neuronal circuits in mammals.

The work also opens up a number of exciting challenges for theory and experiment. Like many behaviours, scratching is a rhythmic action, driven by repeated bursts of spikes. Petersen and Berg focused on the spikes within bursts, but there is silence between bursts. This silence means there must be two timescales for the control of neural activity in the spinal network. Spikes within the bursts arise from fast changes to a neuron's inputs, whether in the fluctuation-driven regime or the mean-driven regime. And the silence between bursts means that the network is able to slowly switch in and out of the driven regimes; that is, it can periodically turn its balanced state on and then off. Clearly we have just started to unpack how neuronal networks control their own activity.
